# Breast cancer but not the menopausal status is associated with small changes of the gut microbiota

**DOI:** 10.3389/fonc.2024.1279132

**Published:** 2024-01-24

**Authors:** Natalia Zeber-Lubecka, Maria Kulecka, Agnieszka Jagiełło-Gruszfeld, Michalina Dąbrowska, Anna Kluska, Magdalena Piątkowska, Katarzyna Bagińska, Maria Głowienka, Piotr Surynt, Michał Tenderenda, Michał Mikula, Jerzy Ostrowski

**Affiliations:** ^1^ Department of Gastroenterology, Hepatology and Clinical Oncology, Centre of Postgraduate Medical Education, Warsaw, Poland; ^2^ Department of Genetics, Maria Sklodowska-Curie National Research Institute of Oncology, Warsaw, Poland; ^3^ Department of Breast Cancer & Reconstructive Surgery, Maria Sklodowska-Curie National Research Institute of Oncology, Warsaw, Poland; ^4^ Department of Oncological Surgery and Neuroendocrine Tumors, Maria Sklodowska-Curie National Research Institute of Oncology, Warsaw, Poland

**Keywords:** breast cancer, gut dysbiosis, microbiome, menopausal status, shotgun

## Abstract

**Background:**

Possible relationships between gut dysbiosis and breast cancer (BC) development and progression have been previously reported. However, the results of these metagenomics studies are inconsistent. Our study involved 88 patients diagnosed with breast cancer and 86 cancer-free control women. Participants were divided into groups based on their menopausal status. Fecal samples were collected from 47 and 41 pre- and postmenopausal newly diagnosed breast cancer patients and 51 and 35 pre- and postmenopausal controls, respectively. In this study, we performed shotgun metagenomic analyses to compare the gut microbial community between pre- and postmenopausal BC patients and the corresponding controls.

**Results:**

Firstly, we identified 12, 64, 158, and 455 bacterial taxa on the taxonomy level of phyla, families, genera, and species, respectively. Insignificant differences of the Shannon index and β-diversity were found at the genus and species levels between pre- and postmenopausal controls; the differences concerned only the Chao index at the species level. No differences in α-diversity indexes were found between pre- and postmenopausal BC patients, although β-diversity differed these subgroups at the genus and species levels. Consistently, only the abundance of single taxa differed between pre- and postmenopausal controls and cases, while the abundances of 14 and 23 taxa differed or tended to differ between premenopausal cases and controls, and between postmenopausal cases and controls, respectively. There were similar differences in the distribution of enterotypes. Of 460 bacterial MetaCyc pathways discovered, no pathways differentiated pre- and postmenopausal controls or BC patients, while two and one pathways differentiated cases from controls in the pre- and postmenopausal subgroups, respectively.

**Conclusion:**

While our findings did not reveal an association of changes in the overall microbiota composition and selected taxa with the menopausal status in cases and controls, they confirmed differences of the gut microbiota between pre- and postmenopausal BC patients and the corresponding controls. However, these differences were less extensive than those described previously.

## Introduction

The healthy adult gut ecosystem is dominated by anaerobic members of the *Firmicutes* and *Bacteroidetes* phyla, and to a lesser extent of the *Actinobacteria, Proteobacteria, Verrucomicrobia*, and *Fusobacteria* phyla (1). The gut microbiota harvests nutrients and energy from the diet, trains the immune system, protects against opportunistic pathogens, and produces metabolites with both local and systemic actions (2). Changes of the composition of the gut microbiota and/or its local distribution, termed dysbiosis, reflect dynamic interactions between the gut microbiota and host variables. These include host genotype, age, sex, lifestyle, diet, physical activity, and sanitation (1, 3–5), and are highly associated with different human pathologies (6, 7). In cancer patients, the intestinal microbiota may modulate metabolism of microbial-derived metabolites and carcinogens, which, in turn, can enhance or diminish the development and progression of gastrointestinal and extra-gastrointestinal neoplasms, including breast cancer (BC) (7–10).

BC is the most common malignant disorder in women. It is linked with the systemic levels of estrogens and other hormones, and is associated with puberty, pregnancy, menopause, hormonal contraceptives, and hormone replacement therapies (11–13). Half of breast carcinomas are diet-associated (14). The gut microbiota can modulate the metabolism of estrogens and obesity-related chronic inflammation; therefore, the composition of the mammary and gut microbiotas is considered to affect the risk of BC (15–17). Consistently, several studies reported a possible relationship between gut dysbiosis and BC development and progression (18–22). The gut microbiome in BC patients is characterized by increased abundances of *Clostridiaceae, Faecalibacterium*, and *Ruminococcaceae*, and decreased abundances of *Dorea* and *Lachnospiraceae*. In addition, the numbers of *Bifidobacterium* and *Blautia* and the proportions of *Faecalibacterium prausnitzii* and *Blautia* vary according to the clinical stage (7, 19).

Changes in the gut microbiota composition have been commonly found across different groups of neoplasms, but the results of metagenomics studies are not always consistent, which makes it difficult to summarize cancer gut dysbiosis. In this study, we performed shotgun sequencing to determine gut microbial signatures associated with pre- and postmenopausal BC patients because menopause alters the gut microbiome (23).

## Materials and methods

### Patients

Between 2018 and 2021, 174 participants were prospectively enrolled in this study, including 88 patients with BC newly diagnosed at the Maria Sklodowska-Curie National Research Institute of Oncology and 86 cancer-free control women. Of them, 47 cases and 51 controls were pre- or perimenopausal (age 27–51 and 18–53 years, respectively), and 41 cases and 35 controls were postmenopausal (age 55–79 and 54–82 years, respectively), according to the STRAW guidelines (24).

As summarized in **Table 1**, the majority of tumors were ductal type (94%). In total, 56 and 32 patients were diagnosed with stage 0–2 and stage 3 BC, respectively. According to immunohistochemical analysis of hormone receptors, 20, 10, 24, 22, and 12 patients had luminal A, luminal B (HER2+ or HER2−), luminal B-like, HER2-enriched, and triple-negative breast cancer (TNBC), respectively. Patients in the pre- and postmenopausal groups had similar body mass indexes. Exclusion criteria included antibiotic use within 2 months before fecal sampling and inflammatory bowel disease for both groups, and a history of cancer for the control group.

**Table 1:** Characteristics of the enrolled patients.

		Pre- or perimenopausal (n=47)	Postmenopausal (n=41)
Stage	0 (in situ)	1	1
	1	3	5
	2	25	21
	3	18	14
Immunohistochemical assay of hormone receptors	Luminal A	9	11
	Luminal B	6	4
	Luminal B-like	15	9
	HER2-enriched	13	9
	Triple negative	4	8
Histologic images	Ductal	42	34
	Ductal carcinoma in situ	2	3
	Lobular	2	3
	Not otherwise specified	1	
	Mixed		1

Fecal samples were collected before systemic cancer treatment. Sixty-nine BC patients were scheduled to receive neoadjuvant systemic therapy, including one of the following: TCH regimen (docetaxel, carboplatin, trastuzumab), TCH-P regimen (docetaxel, carboplatin, trastuzumab, pertuzumab), ACdd regimen (doxorubicin, cyclophosphamide), or paclitaxel treatment. The treatment decision depended on the stage of the disease and the biological subtype of cancer (25).

### Metagenomic sequencing

Genomic bacterial DNA was extracted from 200 mg of feces using a QIAamp Fast DNA Stool Mini Kit (Qiagen, Hilden, Germany) according to the manufacturer’s instructions (26). DNA was quantified using fluorimetry with the Qubit dsDNA High Sensitivity Assay (Thermo Fisher Scientific, Carlsbad, CA, USA). Shotgun metagenomic sequencing was performed using 10 ng of extracted DNA on a NovaSeq 6000 system (Illumina, San Diego, CA, USA) with 100 bp paired-end reads following standard methods provided by the manufacturer (27).

### Bioinformatic analysis

The Shannon and Chao indexes (with confidence intervals) were calculated using the diversity function in the vegan package (version 2.5-7) (28). Values were compared using the Kruskal-Wallis or Mann-Whitney U test (for two groups only). Bacterial taxa were assigned with Metaphlan3 (29), version 3.0.13, using default parameters. Enterotypes were assigned according to the methods described by Arumugam et al. (30), using the R code available at https://enterotype.embl.de/enterotypes.html. Fisher’s exact test was used to verify relationships between experimental groups and enterotypes. *Post-hoc* analysis was performed according to the methods described by Shan and Gerstenberger (31). Differences in taxon abundances between groups were assessed using the LINDA (LInear model for Differential Abundance) (32) method for compositional data, and p-values were corrected using the Benjamini–Hochberg (33) method to minimize the false discovery rate (FDR). Differences in enterotypes were assessed using the Kruskal-Wallis test (with the FDR-corrected Mann-Whitney U *post-hoc* test). Functional assignments were performed using human version 3.0 (part of BioBakery Workflows (29)), using MetaCyc pathways (34) as a reference database. Quality filtering and decontamination were performed with KneadData. The LINDA method was used to assess compositional data, with p-values corrected by the Benjamini–Hochberg procedure to minimize the FDR. Boosted tree models were prepared using the R package xgboost version 1.7.5.1 (35). Models were prepared separately for pre- and postmenopausal women. The dataset was split 7:3 between training and test sets. The model parameters were tuned with grid search [R package caret version 6.0 (36)] using the 10-fold cross-validation procedure. Variable importance plots were prepared with R package SHAPforxgboost (37) [SHAPforxgboost: SHAP Plots for ‘XGBoost’; R package version 0.0.3 (38)]. Receiver operating characteristic curves and values were prepared based on predictions for the test set. All sequencing data are available in SRA at PRJNA1001944 (http://www.ncbi.nlm.nih.gov/bioproject/1001944).

## Results

**Figure 1:**
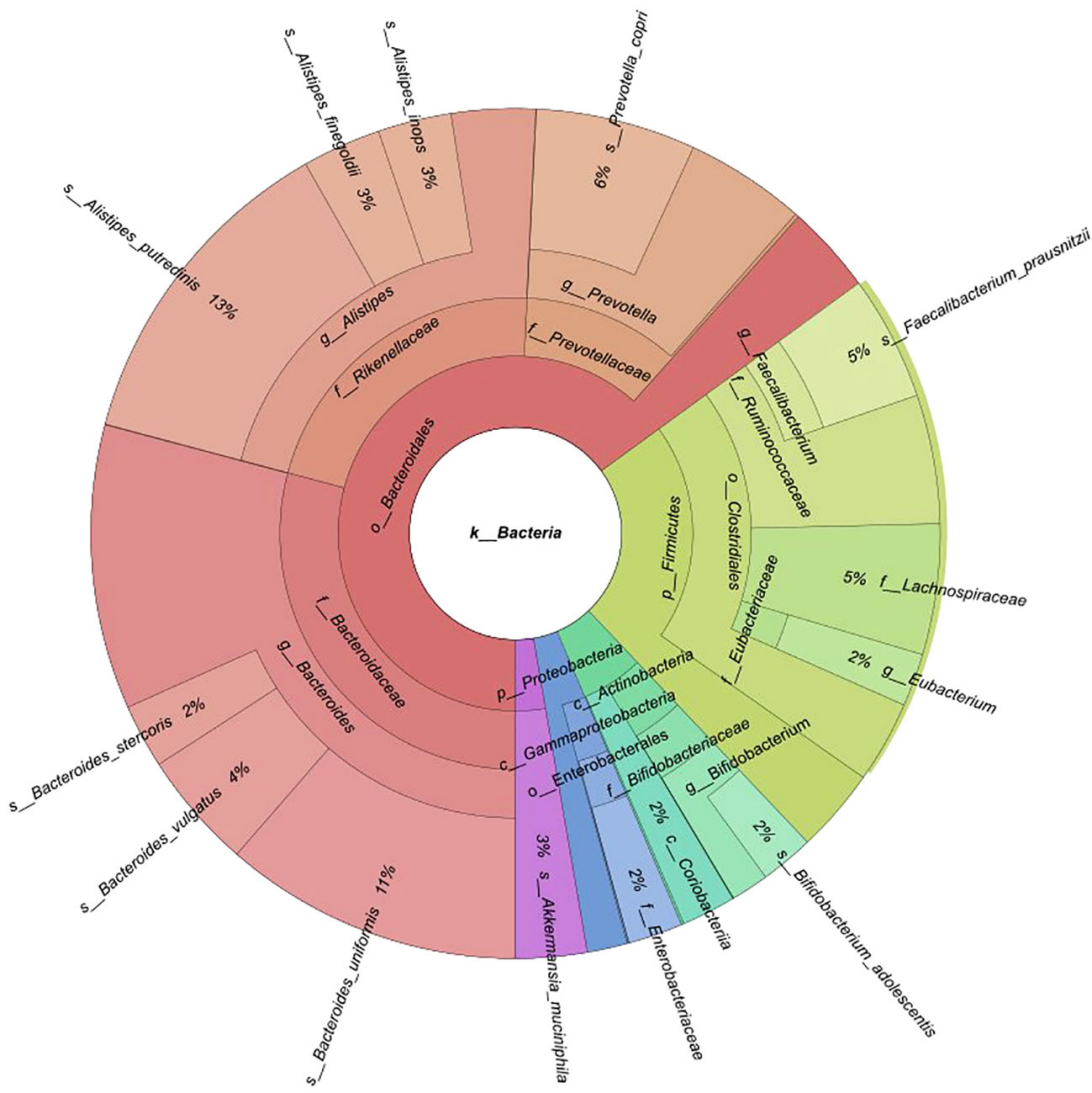
Krona chart of phyla and families with mean abundances greater than 1% of the total.

Fecal samples were collected from 174 participants (88 BC patients and 86 controls). An average of 40 million reads were generated per sample (median 37 million). Of 12 phyla, 64 families, and 158 genera discovered, 5, 15, and 16 had mean abundances greater than 1%, respectively. A total of 455 species were identified. Taxonomic profiling revealed that *Bacteroidetes* were the most abundant phylum (mean 65.1%), followed by *Firmicutes* (mean 23%), *Actinobacteria* (mean 5.7%), *Proteobacteria* (mean 3.6%), and *Verrucomicrobia* (mean 2.7%) (**Figure 1**). The *Firmicutes/Bacteroidetes* (F/B) ratio did not significantly differ between pre- and postmenopausal controls or cases, or between pre- or postmenopausal cases and the corresponding controls (**Supplementary Figure 1**). At the family level, *Bacteroidaceae* (29.2%) and *Rikenellaceae* (22%) [*Bacteroidetes*]; *Ruminococcaceae* (9.7%), *Lachnospiraceae* (5%), and *Eubacteriaceae* (2%) [*Firmicutes*]; *Bifidobacteriaceae* (3.5%) [*Actinobacteria*]; *Enterobacteriaceae* (2.1%) [*Proteobacteria*]; and *Akkermansiaceae* (2.7%) [*Verrucomicrobia*] were most abundant (**Figure 1**).

### Bacterial community structure and enterotype-related changes

To identify potential differences in the structure of the gut microbiome, we first evaluated α- and β-diversity of fecal microbiota in subgroups of cases and controls divided according to their hormonal status. α-diversity was analyzed using the Shannon index, a marker of bacterial richness and evenness, and the Chao index, a marker of bacterial richness. β-diversity was analyzed using principal coordinate analysis (PCoA) of Bray-Curtis distances. The analyses were performed at the genus and species levels.

**Figure 2:**
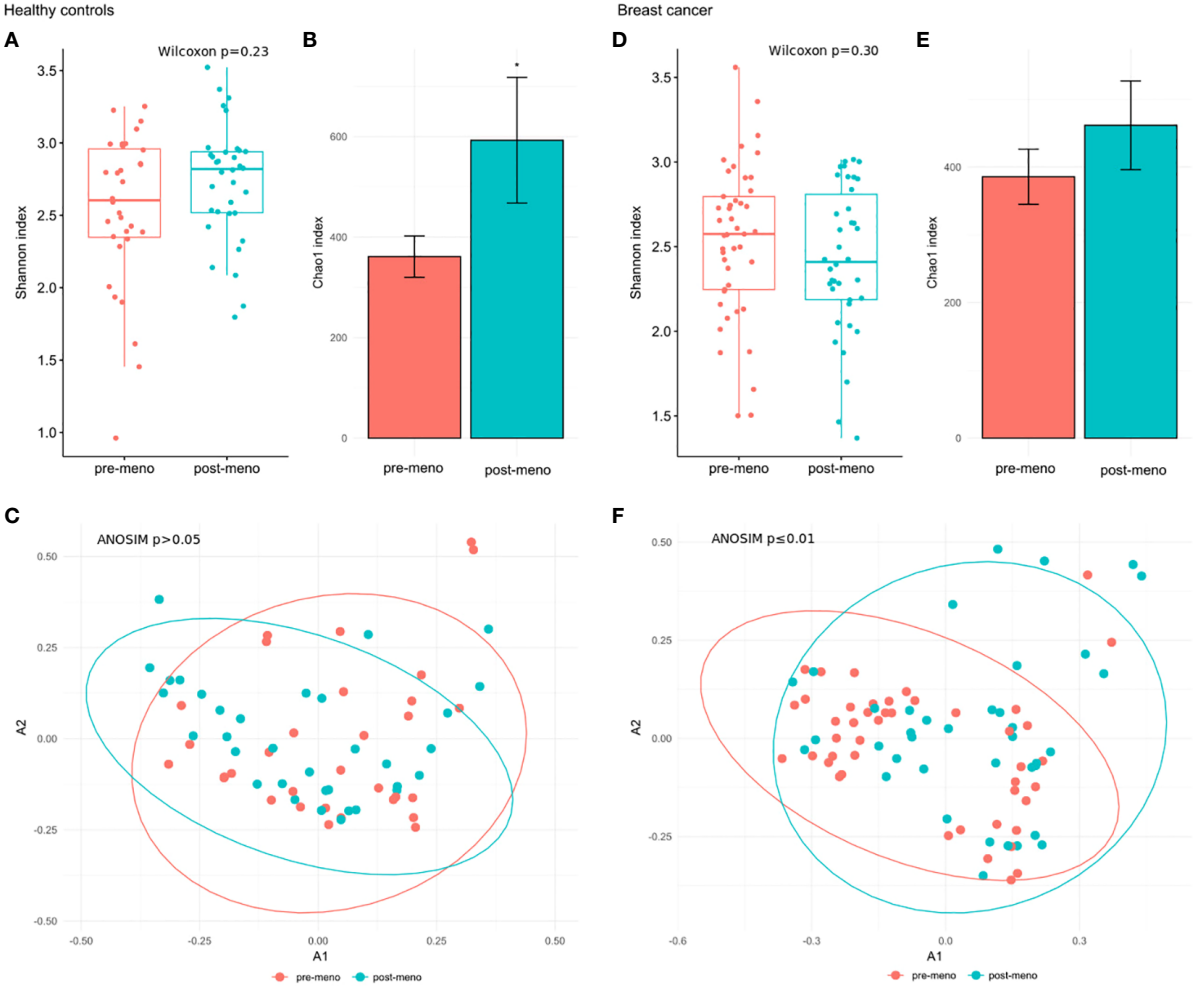
**(A)** Wilcoxon rank-sum for differences in α-diversity (Shannon index) at the species level between pre- and postmenopausal controls. **(B)** Chao index values with 95% confidence intervals at the species level between pre- and postmenopausal controls. The differences of the Chao indexes were considered significant if their confidence intervals do not overlap with reference groups and were marked with asterisk. **(C)** β-diversity measured by analysis of similarity (ANOSIM) using the Bray-Curtis distances at the species level in pre- and postmenopausal controls. **(D)** Wilcoxon rank-sum test for differences in α-diversity (Shannon index) at the species level between pre- and postmenopausal BC patients. **(E)** Chao index values with 95% confidence intervals at the species level between pre- and postmenopausal BC patients. **(F)** β-diversity measured by analysis of similarity (ANOSIM) using the Bray-Curtis distances at the species level in pre- and postmenopausal BC patients.

The gut microbial community structure did not differ between pre- and postmenopausal controls based on insignificant differences in the Shannon index (**Figure 2A**) and β-diversity (**Figure 2C**) at the species and genus levels (**Supplementary Figures 2A, C**). The differences of the Chao indexes were considered significant if their confidence intervals do not overlap with reference groups and were marked with asterisk. For this comparison, the differences concerned only the Chao index at the species level (**Figure 2B**). Similar comparisons of pre- and postmenopausal BC patients revealed no differences in α-diversity indexes at the species level (**Figures 2D, E**), although significant differences in β-diversity were found at the genus (p_adj_ ≤ 0.01) and species (p_adj_ ≤ 0.01) levels (**Supplementary Figure 2F**; **Figure 2F**, respectively).

**Figure 3:**
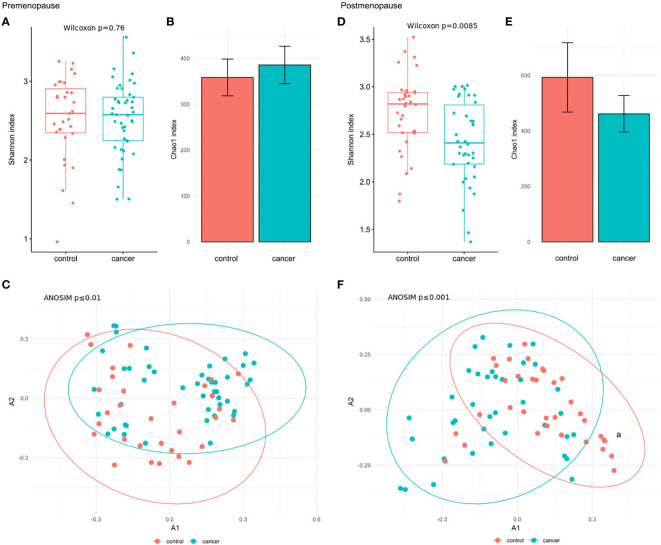
**(A)** Wilcoxon rank-sum test for differences in α-diversity (Shannon index) at the species level in premenopausal BC patients and the corresponding controls. **(B)** Chao index values with 95% confidence intervals at the species level in premenopausal BC patients and the corresponding controls. **(C)** β-diversity measured by analysis of similarity (ANOSIM) using the Bray-Curtis distances at the species level in premenopausal BC patients and the corresponding controls. **(D)** Wilcoxon rank-sum test for differences in α-diversity (Shannon index) at the species level in postmenopausal BC patients and the corresponding controls. **(E)** Chao index values with 95% confidence intervals at the species level in postmenopausal BC patients and the corresponding controls. **(F)** β-diversity measured by analysis of similarity (ANOSIM) using the Bray-Curtis distances at the species level in postmenopausal BC patients and the corresponding controls.

When BC subgroups were compared with the corresponding control subgroup at the species level, the Shannon index was significantly reduced in postmenopausal cases (p_adj_=0.0085) (**Figure 3D**), while no significant changes were observed in premenopausal patients (**Figure 3A**). β-diversity differentiated pre- and postmenopausal cases from the corresponding controls at both the genus (premenopause p_adj_ ≤ 0.001; postmenopause p_adj_ ≤ 0.01) and species levels (premenopause p_adj_ ≤ 0.01; postmenopause p_adj_ ≤ 0.001) (**Supplementary Figures 3C, F**; **Figures 3C, F**, respectively). No differences in the Chao index were observed across pre- and postmenopausal BC subgroup in comparison to controls (**Figures 3B, E**).

**Figure 4:**
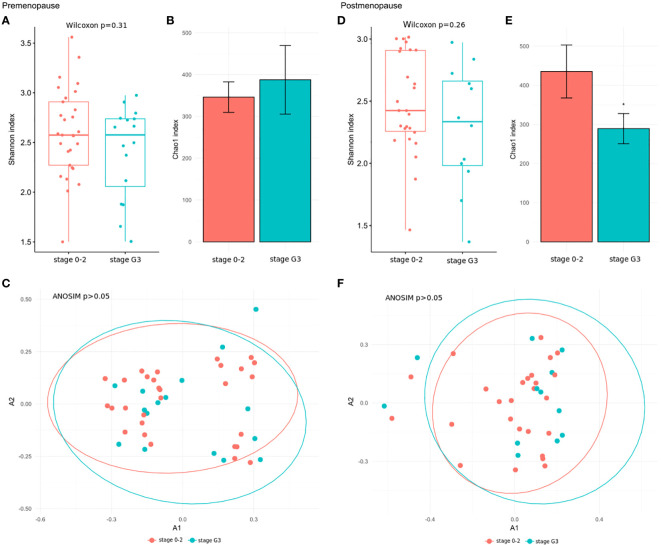
**(A, D)** Wilcoxon rank-sum test for differences in α-diversity (Shannon index) at the species level in comparison between stage 3 and stage 0–2 BC patients in the pre- and postmenopausal subgroups. **(B, E)** Chao index values with 95% confidence intervals at the species level between stage 3 and stage 0–2 BC patients in the pre- and postmenopausal subgroups. The differences of the Chao indexes were considered significant if their confidence intervals do not overlap with reference groups and were marked with asterisk. **(C, F)** Comparison of β-diversity measured by analysis of similarity (ANOSIM) using the Bray-Curtis distances at the species level between stage 3 and stage 0–2 BC patients in the pre- and postmenopausal subgroups.

**Figure 5:**
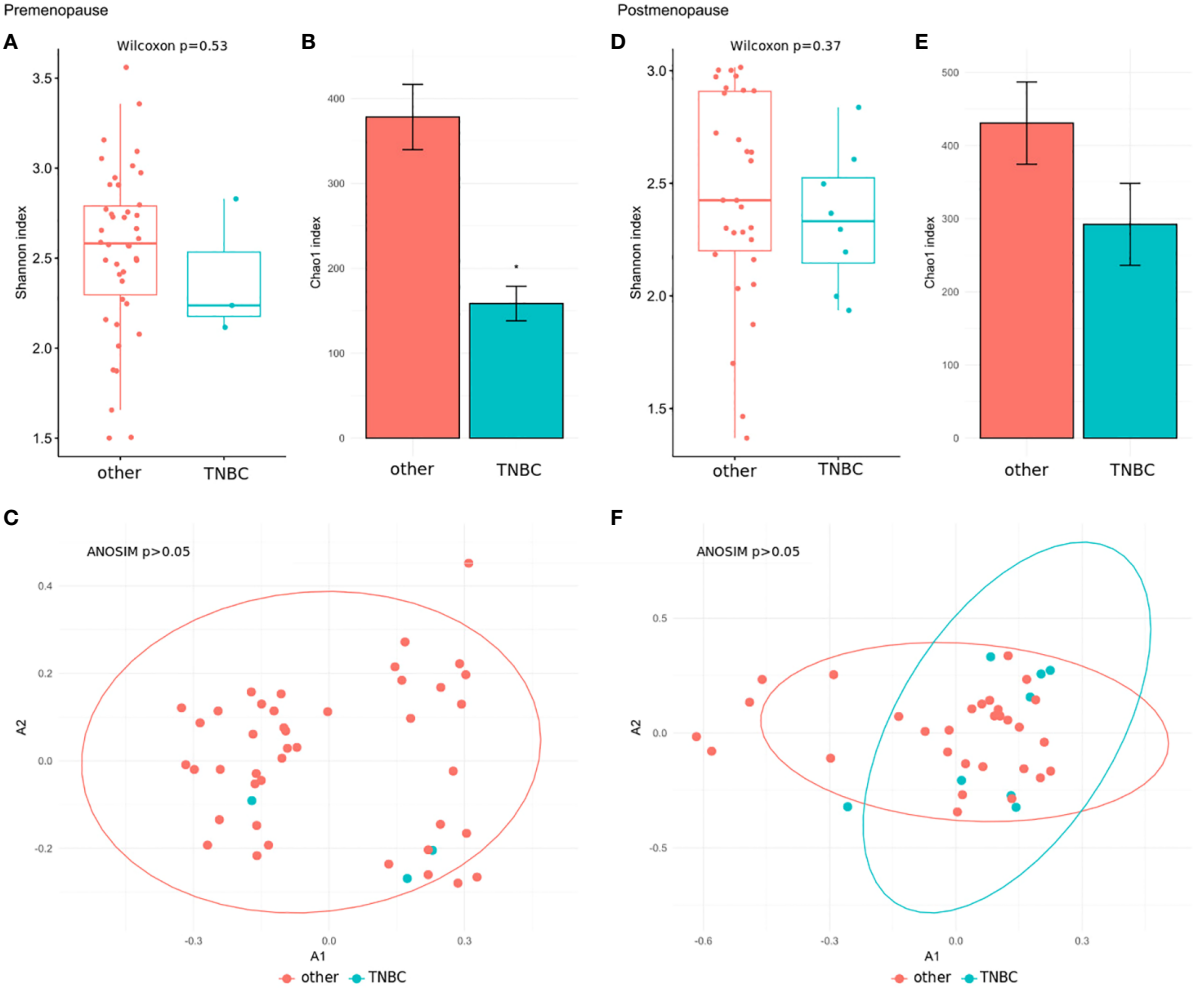
**(A, D)** Wilcoxon rank-sum test for differences in α-diversity (Shannon index) at the species level in comparison between TNBC patients and patients with all other immunohistochemical subtypes in the pre- and postmenopausal subgroups. **(B, E)** Chao index values with 95% confidence intervals at the species level between TNBC patients and patients with all other immunohistochemical subtypes in the pre- and postmenopausal subgroups. The differences of the Chao indexes were considered significant if their confidence intervals do not overlap with reference groups and were marked with asterisk. **(C, F)** β-diversity measured by analysis of similarity (ANOSIM) using the Bray-Curtis distances at the species level in comparison between TNBC patients and patients with all other immunohistochemical subtypes in the pre- and postmenopausal subgroups.

We further explored associations between the community structure of the gut microbiota and pathological indexes. The Shannon index and β-diversity did not significantly differ between stage 3 and stage 0–2 BC patients in the pre- and postmenopausal subgroups (**Figures 4A, C, D, F**). The Chao index was lower in postmenopausal (**Figure 4E**), but not premenopausal, stage 3 BC patients (**Figure 4B**). Similar comparisons of patients with TNBC and those with all other immunohistochemical subtypes detected insignificant differences in the Shannon index (**Figures 5A, D**) and β-diversity (**Figures 5C, F**), but the Chao index was significantly lower in premenopausal TNBC patients (**Figure 5B**) however not in postmenopausal cases (**Figure 5E**).

We grouped controls and BC patients into three and five enterotypes for post- and premenopausal women, respectively. In both post- and premenopausal women, *Bacteroides-*dominated enterotypes were overrepresented in controls, while *Prevotella*- and *Alistipes*-based enterotypes were overrepresented in BC patients (**Table 2**).

**Table 2:** Distribution of enterotypes in pre- and postmenopausal women revealed by Fisher’s exact test.

Premenopause					
	1 *Bacteroides*	2 *Alistipes*	3 *Prevotella*	4 *Akkermansia*	5 *Oscillibacter*
Controls	17 (55%)*	5 (17%)*	1 (3%)	2 (6%)	6 (20%)
BC cases	10 (22%)*	20 (44%)*	5 (11%)	1 (3%)	9 (20%)
Postmenopause					
			1 *Bacteroides*	2 *Prevotella*	*3 Alistipes*
Controls			18 (53%)*	6 (18%)*	10 (29%)
BC cases			10 (26%)*	16 (42%)*	12 (32%)

*,statistically significant at p<0.05; %, percentage distribution of enetrotype.

### Bacterial abundances

P-values <0.05 and <0.1 after adjustment for multiple comparisons indicated that the relative abundances of taxa were significantly different or tended to be different, respectively. Comparisons between subgroups of pre- and postmenopausal controls and cases showed only single potentially differing taxa. The phylum *Synergistetes* tended to be overrepresented (log2FC=1.30; p_adj_=0.078), and the genera *Allisonella* and *Bifidobacterium* tended to be underrepresented (log2FC=0.76 and -2.18; p_adj_=0.059 and 0.058) in postmenopausal controls and cases, respectively. By contrast, the abundances of 14 taxa related to phyla, families, genera, or species differed or tended to differ between premenopausal cases and premenopausal controls (**Table 3**). All of them except the genus *Coprobacter* had lower abundances in BC patients. In postmenopausal cases, 15 and 8 taxa were under- and overrepresented, respectively, compared with postmenopausal controls (**Table 4**).

**Table 3:** Taxa differentiating premenopausal BC patients from premenopausal controls assessed using the LInear model for Differential Abundance (LINDA) method for compositional data, and p-values were corrected using the Benjamini–Hochberg.

Taxa	baseMean	log2FC	lfcSE	p_adj_
*s:Collinsella_massiliensis*	97.40	2.32	0.62	0.026
*s:Gemmiger_formicilis*	2633.12	1.94	0.51	0.026
*s:Collinsella_stercoris*	266.09	1.68	0.50	0.069
*g:Gemmiger*	964.44	2.20	0.52	0.005
*g:Bifidobacterium*	10046.86	2.82	0.72	0.007
*g:Ruthenibacterium*	2621.61	1.96	0.54	0.012
*g:Anaeromassilibacillus*	26.48	2.52	0.80	0.044
*g:Enorma*	64.10	2.03	0.72	0.082
*g:Coprobacter*	362.33	-2.11	0.76	0.082
*f:Bifidobacteriaceae*	9212.46	2.89	0.71	0.003
*f:Coriobacteriaceae*	9741.54	1.49	0.52	0.005
*f:Eggerthellaceae*	1823.20	1.62	0.59	0.007
*f:Actinomycetaceae*	8.04	1.89	0.71	0.009
*p:Actinobacteria*	28231.02	1.17	0.38	0.002

baseMean, the average of the normalized count values, divided by size factors, taken over all samples; log2FC, log2 fold change between the groups; lfcSE, standard error of the log2FC estimate; p_adj_, Benjamini–Hochberg-adjusted p-value; s, species; g, genus; f, family; p, phylum.

Results with Benjamini–Hochberg-adjusted p-value <0.05 were identified as statistically significant.

**Table 4:** Taxa differentiating postmenopausal BC patients from postmenopausal controls assessed using the LInear model for Differential Abundance (LINDA) method for compositional data, and p-values were corrected using the Benjamini–Hochberg.

Taxa	baseMean	log2FC	lfcSE	p_adj_
*s:Coprobacter_fastidiosus*	489.37	-3.80	0.89	0.011
*s:Collinsella_intestinalis*	19.71	1.82	0.54	0.036
*s:Bacteroides_thetaiotaomicron*	6710.67	-2.47	0.71	0.036
*s:Parabacteroides_distasonis*	32489.88	-1.81	0.53	0.036
*s:Blautia_obeum*	670.59	-2.11	0.60	0.036
*s:Phascolarctobacterium_faecium*	784.05	-3.45	0.96	0.036
*s:Clostridium_sp_CAG_167*	731.61	-1.55	0.51	0.080
*s:Barnesiella_intestinihominis*	18725.04	-2.82	0.94	0.086
*s:Dorea_formicigenerans*	423.54	-2.22	0.77	0.092
*s:Agathobaculum_butyriciproducens*	1446.39	1.88	0.65	0.092
*g:Coprobacter*	259.57	-3.91	0.93	0.005
*g:Parabacteroides*	33034.11	-1.74	0.55	0.036
*g:Dorea*	1936.61	-2.18	0.64	0.036
*g:Agathobaculum*	615.57	2.04	0.63	0.036
*g:Harryflintia*	72.23	0.96	0.30	0.036
*g:Enterorhabdus*	5.41	1.39	0.47	0.053
*g:Rothia*	28.07	1.32	0.46	0.055
*g:Blautia*	2304.17	-1.52	0.54	0.055
*g:Barnesiella*	8130.01	-2.69	0.97	0.058
*g:Allisonella*	3.95	0.97	0.37	0.074
*g:Bacteroides*	555836.30	-1.33	0.51	0.075
*f:Micrococcaceae*	23.47	1.58	0.43	0.014
*f:Barnesiellaceae*	10883.07	-2.93	0.88	0.025

baseMean, the average of the normalized count values, divided by size factors, taken over all samples; log2FC, log2 fold change between the groups; lfcSE, standard error of the log2FC estimate; p_adj_, Benjamini–Hochberg-adjusted p-value; s, species; g, genus; f, family. Results with Benjamini–Hochberg-adjusted p-value <0.05 were identified as statistically significant.

Three species tended to be underrepresented in premenopausal cases with stage 3 BC compared with other premenopausal BC patients (**Table 5**), but no taxa differentiated TNBC patients from all other BC patients.

**Table 5:** Taxa differentiating premenopausal patients with stage 3 BC from other premenopausal BC patients assessed using the LInear model for Differential Abundance (LINDA) method for compositional data, and p-values were corrected using the Benjamini–Hochberg.

Taxa	baseMean	log2FC	lfcSE	p_adj_
*s:Adlercreutzia_equolifaciens*	462.24	-3.55	1.03	0.067
*s:Asaccharobacter_celatus*	916.51	-3.06	0.83	0.067
*s:Firmicutes_bacterium_CAG_110*	1442.88	-4.94	1.39	0.067

baseMean, the average of the normalized count values, divided by size factors, taken over all samples; log2FC, log2 fold change between the groups; lfcSE, standard error of the log2FC estimate; p_adj_, Benjamini–Hochberg-adjusted p-value; s, species.

Results with Benjamini–Hochberg-adjusted p-value <0.05 were identified as statistically significant.

**Figure 6:**
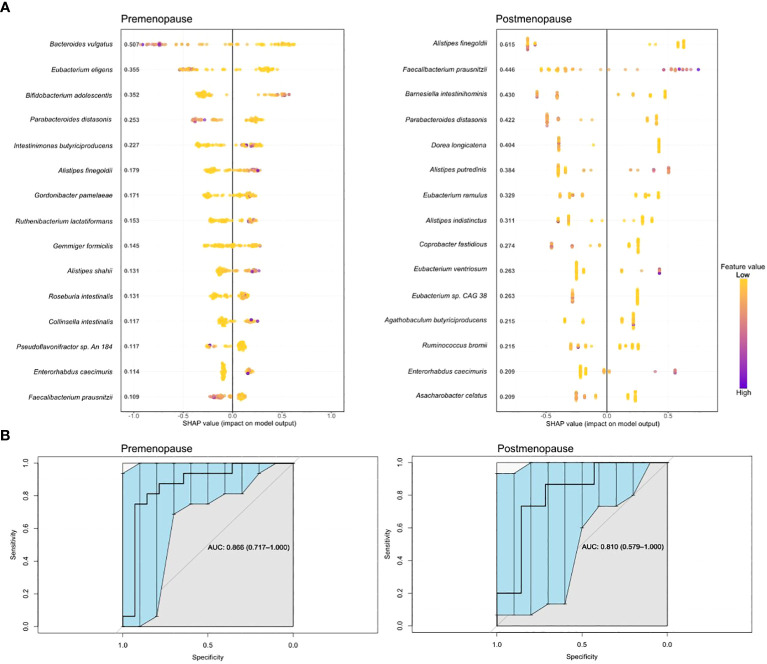
Boosted trees machine learning models **(A)** and AUCs **(B)** based on gut microbiome signatures in pre- and postmenopausal BC patients compared with the corresponding controls.

Boosted trees machine learning models based on the gut microbiome signature revealed a potential diagnostic value of the gut microbiota for distinguishing pre- and postmenopausal BC patients from the corresponding controls (**Figure 6A**) with an area under the receiver operating curve (AUC) of 0.866 (95% CI: 0.717–1.000) and 0.810 (95% CI: 0.579–1.000) for the test datasets (**Figure 6B**). Only four bacteria were shared among the top 15 most distinguishing features in both models: *F. prausnitzii, Alistipes finegoldii, Parabacteroides distasonis*, and *Enterorhabdus caecimuris.* Among these bacteria, only *E. caecimuris* had a high importance value in both pre- and postmenopausal BC patients compared with controls. By contrast, *P. distasonis* had a high importance value in pre- and postmenopausal controls compared with BC patients. *A. finegoldii* and *F. prausnitzii* had high importance values in pre- and postmenopausal BC patients, respectively.

### Functional analysis

A total of 460 bacterial MetaCyc pathways were discovered. No pathways differentiated pre- and postmenopausal controls or BC patients. Two and one pathways differentiated BC patients from controls in the pre- and postmenopausal groups, respectively. Another two and one pathways exhibited differential trends (**Table 6**).

**Table 6:** Pathways differentiating BC patients from controls performed using human version 3.0 (part of BioBakery Workflows, using MetaCyc pathways as a reference database.

	Premenopausal BC patients *vs.* controls			
	baseMean	log2FC	lfcSE	p_adj_
*NAD-BIOSYNTHESIS-II: NAD salvage pathway II*	374.75	-1.76	0.42	0.015
*P461-PWY: hexitol fermentation to lactate, formate, ethanol, and acetate*	305.42	1.20	0.29	0.015
*POLYISOPRENSYN-PWY: polyisoprenoid biosynthesis (E. coli)*	974.06	-0.98	0.28	0.069
*PWY-6859: all-trans-farnesol biosynthesis*	645.99	-1.16	0.32	0.069
	Postmenopausal BC patients vs. controls			
	baseMean	log2FC	lfcSE	p_adj_
*PWY-5177: glutaryl-CoA degradation*	978.20	0.72	0.17	0.039
*PWY-7316: dTDP-N-acetylviosamine biosynthesis*	82.36	-1.99	0.53	0.073

baseMean, the average of the normalized count values, divided by size factors, taken over all samples; log2FC, log2 fold change between the groups; lfcSE, standard error of the log2FC estimate; p_adj_, Benjamini–Hochberg-adjusted p-value.

Results with Benjamini–Hochberg-adjusted p-value <0.05 were identified as statistically significant.

## Discussion

Development and progression of BC can be enhanced or diminished by estrogen-dependent and -independent dynamic interactions between the gut ecosystem, which comprises at least 1800 genera and 15,000–36,000 bacterial species (39, 40), and host variables. A subset of the gut microbiota, referred to as the estrobolome, has been linked to a high level of circulating estrogens in postmenopausal estrogen receptor (ER)-positive BC patients, while a diet, which is one of the strongest modulators of the gut microbiota, enriched with mono- and polyunsaturated fatty acids, fruits, vegetables, and legumes may reduce BC risk and mortality (14, 41).

In this metagenomic study, we performed shotgun sequencing of bacterial DNA isolated from fecal samples to determine changes in the gut microbiota composition among newly diagnosed Polish BC patients and comparable cancer-free controls, divided into pre- and postmenopausal subgroups. Within-sample (α) diversity of the richness and evenness of the gut microbiota was estimated based on the Shannon and Chao indexes. Inter-sample (β) diversity was estimated based on PCoA of Bray-Curtis distances.

The Shannon index did not significantly differ between pre- and postmenopausal controls at the genus and species levels, while the Chao index was lower in premenopausal controls than in postmenopausal controls. These results are consistent with those of Zhu et al. (21), who reported that the gut microbial community structure in healthy women does not depend on the menopausal status, but are inconsistent with those of two other studies, which reported that α-diversity is lower in healthy postmenopausal women (42). Consistent with a study by Hou et al. (43), we found that the Shannon index was significantly reduced in postmenopausal BC patients compared with matched controls. A study by Aarnoutsein et al. (44) found that microbial richness and diversity did not significantly differ between postmenopausal ER+/HER2- BC patients and controls, while a case-control study by Byrd et al. (45) conducted in a Ghanian population found that α-diversity was significantly lower in cases than in controls, but did not differ between BC and non-malignant cases. In summary, we and others [18] [48] detected more evident changes in gut microbiota richness using the Chao index than the Shannon index, which determines both richness and evenness.

β-diversity is strongly and similarly associated with BC and non-malignant breast disease (46). In our study, β-diversity differentiated both pre- and postmenopausal cases from the corresponding controls at the genus and species levels. In addition, enterotype-related changes analyzed at the genus level differentiated both pre- and postmenopausal cases from controls. However, enterotype frequencies did not differentiate premenopausal controls from postmenopausal controls or premenopausal cases from postmenopausal cases.

Although previous studies suggested that the microbiome in BC patients significantly varies according to clinicopathological grouping, we did not find significant associations of BC staging or immunopathological characteristics with α-diversity measured by the Shannon index or β-diversity. Instead, the Chao index differed according to BC staging in the postmenopausal subgroup and differentiated TNBC patients from those with other hormone receptor statuses in the premenopausal subgroups.

Our taxon-dependent analyses were conducted at the phylum, family, genus, and species levels. We found that the phylum *Synergistetes* tended to be overrepresented in fecal samples from postmenopausal controls. He et al. (47) reported that the abundance of *Synergistetes* was significantly higher in the postmenopausal osteopenia group than in the control group, and Soliman et al. (48) showed that bacterial OUTs (operational taxonomic units) belonging to the phylum *Synergistetes* were higher in women who did not receive hormone replacement therapy during menopause. However, the differences in relative taxa levels between pre- and postmenopausal controls or BC patients were weak in our study. Instead, the abundances of 14 and 23 taxa differentiated pre- and postmenopausal cases from the corresponding controls, respectively. By performing shotgun sequencing, Zhu et al. (21) did not find significant differences in gut microbiota species between premenopausal BC patients and controls, while 38 species were enriched and seven species were reduced in postmenopausal BC patients compared with controls. By performing 16S sequencing, Hou et al. (43) found 167 OTUs (operational taxonomic units) belonging to premenopausal BC patients versus premenopausal controls, and 232 OTUs belonging to postmenopausal BC patients versus postmenopausal controls. Therefore, there were fewer differentiating taxa in our study than in other studies.

*Bifidobacterium* genera are well-known probiotics related to the maintenance of human health whose abundances decrease with aging. *Bifidobacterium* may modulate inflammation, the immune response, and metabolism, which influence cancer development. Some studies found that higher levels of *Bifidobacterium* in the gut microbiome may be associated with a reduced risk of BC (49). In our study, the abundance of *Bifidobacterium* was reduced in postmenopausal cases, while the abundances of the *Bifidobacterium* genus and *Bifidobacteriaceae* were higher in premenopausal cases than in the corresponding controls. Our results are consistent with those of Liu et al. (50), who reported deficiencies of *Bifidobacterium animalis*, *Aggregatibacter segnis*, and *Acinetobacter guillouiae* in women with menopausal syndrome. By contrast, Hou et al. (43) reported that the abundance of *Bifidobacterium* species was reduced in premenopausal, but not postmenopausal, BC patients. Therefore, the potential role of *Bifidobacteria* in the development or progression of BC requires further research.

In our study, the abundances of the *Blautia* genus and *Blautia obeum* species were lower in postmenopausal cases than in controls. This taxa is involved in metabolism of enterolignans in the human intestine and consequently in phytoestrogen metabolism (51). Thus, metabolism of enterolignans may affect hormone-dependent BC (20). Luu et al. (20) reported that the abundance of the *Blautia* genus is increased in early-stage BC with severe clinical and histoprognostic grades.

A previous study (21) reported several other species in the gut microbiota whose relative abundances differentiated postmenopausal BC patients from controls. Of these, *Escherichia coli*, *Citrobacter koseri*, *Acinetobacter radioresistens*, *Enterococcus gallinarum*, *Shewanella putrefaciens*, *Erwinia amylovora*, *Actinomyces* sp. *HPA0247*, *Salmonella enterica*, *Fusobacterium nucleatum*, and *Prevotella amnii* were overrepresented in BC patients, while *Eubacterium eligens* and *Roseburia inulinivorans* were underrepresented. In a study comparing the gut microbial profiles of pre- and postmenopausal BC patients (43), 14 microbial markers were associated with the menopausal status. Of these, *Bacteroides fragilis* and *Klebsiella pneumoniae* were specifically enriched in young and older patients, respectively, while *Faecalibacterium* and *Bifidobacterium* were enriched in premenopausal controls compared with postmenopausal controls.

Bacteria may be involved in the development of BC through multiple pathways, including those related and unrelated to hormonal regulation (45). Gut taxa containing β-galactosidase are associated both positively (*Ruminococcaceae* and *Bacteroides*) and negatively (*Eubacterium coprostanoligenes* group, *Coprococcus*, *Dorea*, *Collinsella*, *Faecalibacterium*, and *Prevotella*) with breast diseases (45). Other bacteria (e.g., *Faecalibacterium*, *Prevotella*, and family *Ruminococcaceae*) associated with BC and non-malignant breast diseases have been suggested to be markers of systemic inflammation (52). Some other species within the breast disease-associated taxa (e.g., *Bacteroides*, *Dialister*, *Coprococcus, Faecalibacterium*, *Pseudobutyrivibrio*, and *Romboutsia*) may affect gut barrier integrity (45).

Decreases of progesterone and estrogen levels during the late peri- and menopausal periods affect intestinal barrier permeability, which, in turn, increases the translocation of lipopolysaccharide and triggers immune cells to produce pro-inflammatory cytokines (53). In postmenopausal women, the relative abundances of *Firmicutes* and *Bacteroidetes* increase and decrease, respectively, leading to a significantly higher F/B ratio (54). The F/B ratio did not significantly differ in any of our comparisons (data not shown), although the abundances of *Bacteroides thetaiotaomicron* and *Clostridium* sp. *CAG 167* species and the *Bacteroides* genus were significantly lower in postmenopausal BC patients than in controls. *E. coli, Bacteroides* species, and *Clostridium* species are predominant among species that contain β-glucuronidase and sulfatase enzymes, which deconjugate glucuronide and sulfate groups, respectively. Reductions in the abundances of *Bacteroides* and *Clostriudium* species in combination with a decrease of bacterial α-diversity allow for suggesting that gut dysbiosis of postmenopausal BC patients is related to the hormone status rather than to carcinogenesis itself.

In addition to determining differential bacteria in single comparisons between groups, we also determined a predictive model that allowed the identification of species with diagnostic potential in BC in relation to hormone status. *E. caecimuris* had a high importance value in pre- and postmenopausal BC patients compared with the corresponding controls. Bawaneh et al. (55) reported that the proportional abundance of *E. caecimuris* was increased in the microbiome of TNBC patients treated with doxorubicin. In the model, *F. prausnitzii* had a high importance value in premenopausal controls and postmenopausal BC cases. *F. prausnitzii* is underrepresented in lung cancer and BC (56, 57). These data are similar to those reported by Ma et al. in relation to BC (58). Regarding our results, postmenopausal BC patients with a higher abundance of *F. prausnitzii* may have a better prognosis.

TNBC and triple-positive BC have distinct microbial patterns (59, 60). Whereas our study did not confirm differences in the microbiome composition according to the cancer type, three species (*Adlercreutzia equolifaciens*, *Asaccharobacter celatus*, and *Firmicutes bacterium CAG 110*) tended to be underrepresented in premenopausal stage 3 BC patients. A lack of differences in α-diversity according to tumor stage and grade was reported previously (59).

Research of the relationship between intestinal bacteria, menopause, and BC is ongoing, and the specific mechanisms are not fully understood. However, there is evidence that the gut microbiome may play a role in BC development and progression, and hormonal changes during menopause can potentially influence this relationship.

To summarize our results, the richness, diversity, and composition of the gut microbiota did not markedly differ between pre- and postmenopausal BC patients or controls. Therefore, we did not confirm that the gut bacterial community structure in our cases and controls depends on age and menopause status. Instead, gut bacterial dysbiosis, including taxonomic differences, was observed in both pre- and postmenopausal BC patients compared with the corresponding controls.

Most microbial studies of BC have focused on microbiota functions in breast glands and the milk microbiome (61). However, there is limited evidence that certain alterations of metabolite production by the gut microbiota can promote carcinogenesis in organs that are distal to the gut, such as the breasts. For instance, Kovacs et al. (62) discovered that cadaverine metabolite (63) inhibits cancer cell invasion, and bacterial production of cadaverine seems to be reduced in early-stage cancer patients, as evidenced by lower abundances of *CadA* and *LdcC*, which are involved in cadaverine production. Litocholic acid is another gut metabolite that reduces BC cell proliferation (64, 65). In our study, only moderate functional alterations of the gut microbiome were uncovered; two and one pathways differentiated BC patients from controls in the pre- and postmenopausal groups, respectively, while the same numbers of pathways exhibited differential trends. The *NAD salvage* pathway was downregulated in the gut microbiome of premenopausal BC patients. NAD is an important cofactor for many metabolic reactions, including DNA repair (66), and the bacterial NAD salvage pathway is crucial for boosting NAD metabolism in the host (67). Another pathway, *hexitol fermentation to lactate, formate, ethanol, and acetate*, was upregulated in premenopausal BC patients. This pathway is associated with non-response to chemotherapy in lung cancer (68); however, further mechanistic studies are needed to determine its role in carcinogenesis. The *glutaryl-CoA degradation* pathway, which was overrepresented in postmenopausal BC patients, is downregulated in high-risk adenoma patients (69). In summary, functional alterations of the gut microbiome were minimal in our BC patients and it is difficult to discern any direct links between these alterations and carcinogenesis.

The human microbiome is highly individualized, and quantification of inter-sample variations between individuals depends on the occurrence and relative abundances of microbial taxa across multiple samples, and results may depend on the sequencing method used. Thus, sequencing remains an important consideration in metagenomic studies (70). Sequencing of the bacterial 16S rRNA gene detects only part of the gut microbiome community determined by shotgun sequencing. Shotgun sequencing has greater power than 16S sequencing to identify less abundant taxa, which are biologically relevant (71), by ascertaining the bacterial details at the species, gene, and function levels. In this regard, deep shotgun sequencing was a strength of our study. The limitations of this study include a lack of information about certain potential gut dysbiosis confounders, such as lifestyle, diet, and probiotic use.

In conclusion, while our findings did not reveal an association of changes in the overall microbiota composition and selected taxa with the menopausal status in cases and controls, they confirmed differences in the gut microbiota between pre- and postmenopausal cases and the corresponding controls. However, our findings are not completely consistent with those reported previously, and the magnitude of the differences was lower than that of those described before. Previous studies were conducted using different populations, including American, European, and Asian populations, and it is possible that differences in taxa-disease associations, as summarized above, are related to geographic location (72). Future prospective studies should be conducted with more diverse populations and consider variable confounders, such as lifestyle, dietary habits, smoking status, and alcohol consumption, as well as the general status of “healthy” controls, which may actively modulate the gut microbiota structure.

## Data availability statement

The datasets presented in this study can be found in online repositories. The names of the repository/repositories and accession number(s) can be found below: https://www.ncbi.nlm.nih.gov/, PRJNA1001944.

## Ethics statement

The studies involving humans were approved by Bioethics Committee at the Maria Sklodowska-Curie National Research Institute of Oncology (40/2018/1/2019). The studies were conducted in accordance with the local legislation and institutional requirements. The participants provided their written informed consent to participate in this study.

## Author contributions

NZ-L: Investigation, Project administration, Visualization, Writing – original draft, Writing – review & editing. MK: Data curation, Formal analysis, Visualization, Writing – original draft, Writing – review & editing. AJ-G: Resources, Writing – review & editing. MD: Investigation, Writing – review & editing. AK: Investigation, Writing – review & editing. MP: Investigation, Writing – review & editing. KB: Investigation, Writing – review & editing. MG: Investigation, Writing – review & editing. PS: Investigation, Writing – review & editing. MT: Resources, Writing – review & editing. MM: Visualization, Writing – review & editing. JO: Conceptualization, Funding acquisition, Project administration, Supervision, Writing – original draft, Writing – review & editing.

## Supplementary material

Supplementary Figure 1 Wilcoxon rank-sum test for differences in F/B ratios in pre- and postmenopausal controls and BC patients.

Supplementary Figure 2
**(A)** Wilcoxon rank-sum test for differences in α-diversity (Shannon index) and **(B)** Chao index values with 95% confidence intervals at the genus level in pre- and postmenopausal controls. **(C)** β-diversity measured by analysis of similarity (ANOSIM) using the Bray-Curtis distances at the genus level in pre- and postmenopausal controls. **(D)** Wilcoxon rank-sum test for differences in α-diversity (Shannon index) and **(E)** Chao index values with 95% confidence intervals at the genus level in pre- and postmenopausal BC patients. **(F)** β-diversity measured by analysis of similarity (ANOSIM) using the Bray-Curtis distances at the genus level in pre- and postmenopausal BC patients.

Supplementary Figure 3
**(A, D)** Wilcoxon rank-sum test for differences in α-diversity (Shannon index) and **(B, E)** Chao index values with 95% confidence intervals at the genus level in pre- and postmenopausal BC patients and the corresponding controls. **(C, F)** β-diversity measured by analysis of similarity (ANOSIM) using the Bray-Curtis distances at the genus level in pre- and postmenopausal BC patients and the corresponding controls.
