# Supplementary figures and images for: Breast cancer but not the menopausal status is associated with small changes of the gut microbiota
Source: Front Oncol. 2024 Jan 24;14:1279132. doi: 10.3389/fonc.2024.1279132 (PMC10848918; doi:10.3389/fonc.2024.1279132)

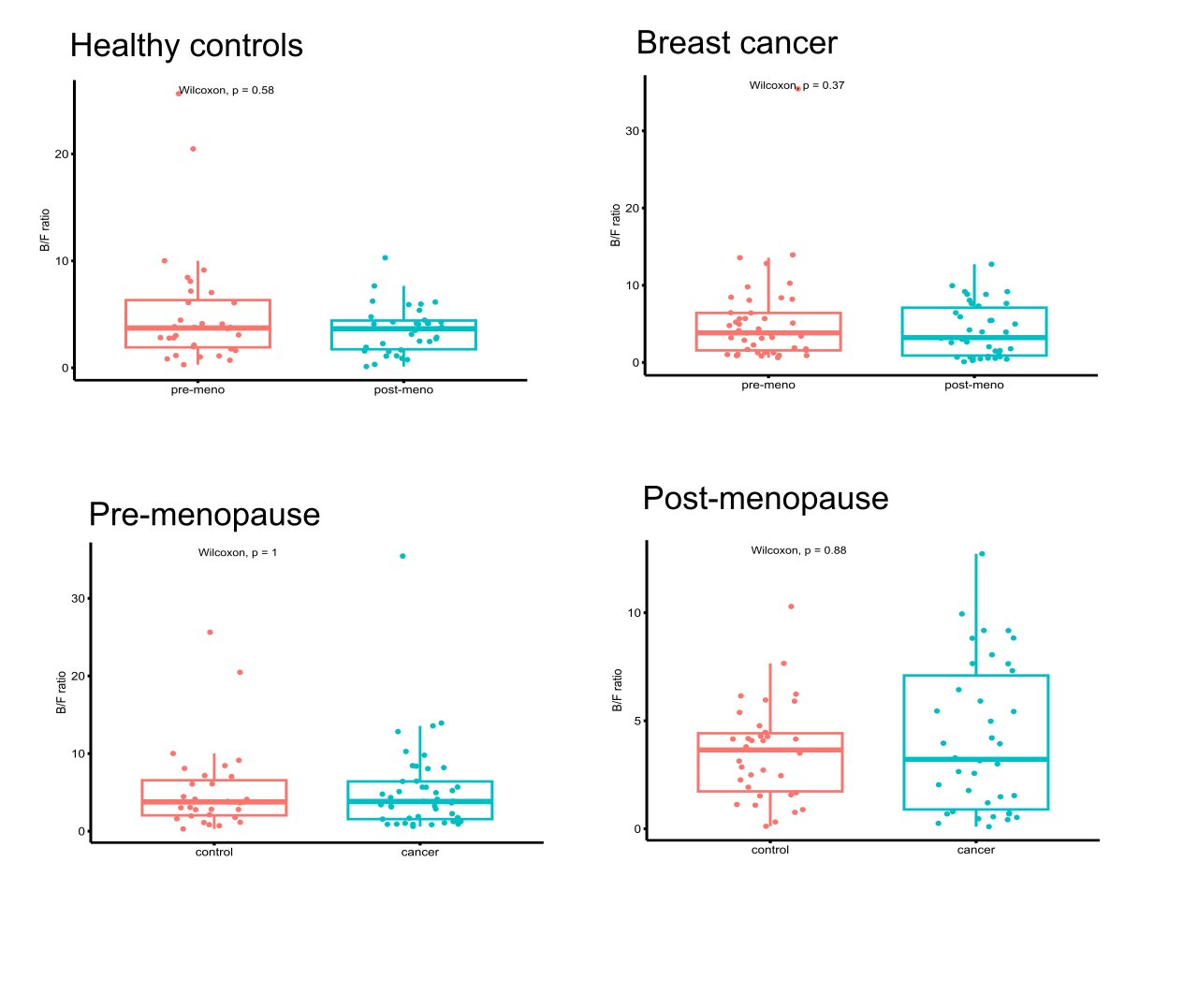

Supplement: Supplementary Figure 1 — Wilcoxon rank-sum test for differences in F/B ratios in pre- and postmenopausal controls and BC patients. [file Image_1.jpeg]

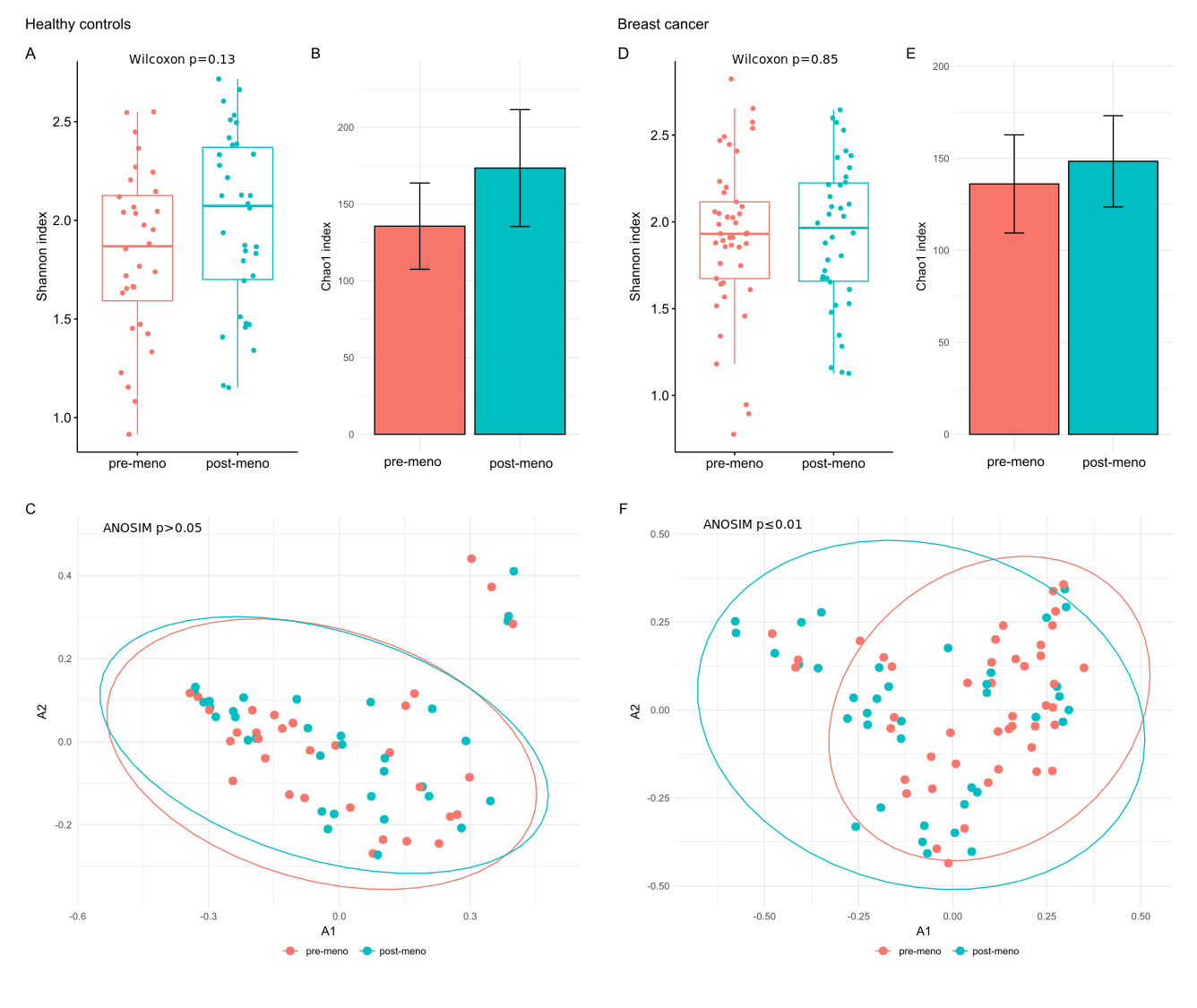

Supplement: Supplementary Figure 2 — (A) Wilcoxon rank-sum test for differences in α-diversity (Shannon index) and (B) Chao index values with 95% confidence intervals at the genus level in pre- and postmenopausal controls. (C) β-diversity measured by analysis of similarity (ANOSIM) using the Bray-Curtis distances at the genus level in pre- and postmenopausal controls. (D) Wilcoxon rank-sum test for differences in α-diversity (Shannon index) and (E) Chao index values with 95% confidence intervals at the genus level in pre- and postmenopausal BC patients. (F) β-diversity measured by analysis of similarity (ANOSIM) using the Bray-Curtis distances at the genus level in pre- and postmenopausal BC patients. [file Image_2.jpeg]

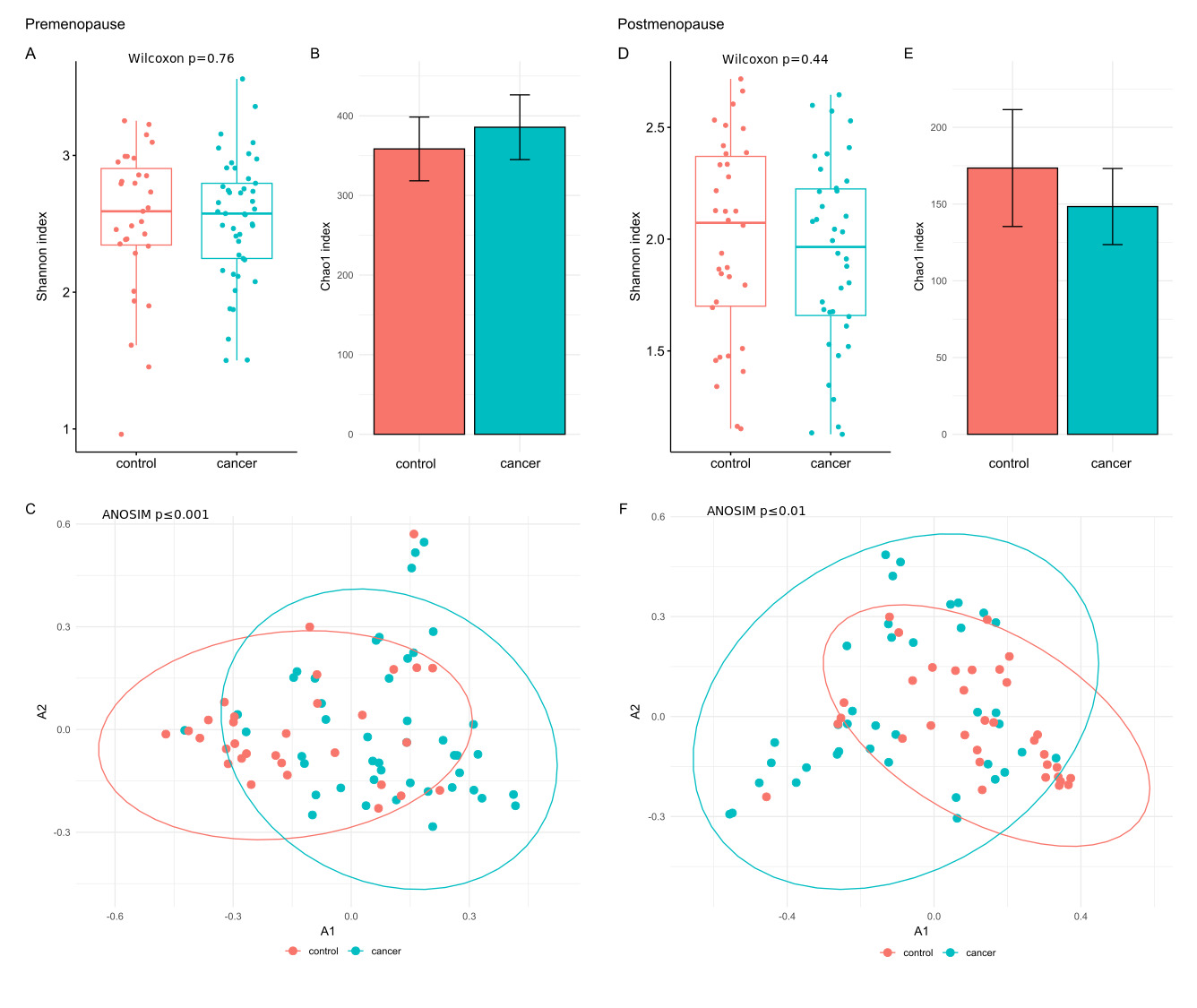

Supplement: Supplementary Figure 3 — (A, D) Wilcoxon rank-sum test for differences in α-diversity (Shannon index) and (B, E) Chao index values with 95% confidence intervals at the genus level in pre- and postmenopausal BC patients and the corresponding controls. (C, F) β-diversity measured by analysis of similarity (ANOSIM) using the Bray-Curtis distances at the genus level in pre- and postmenopausal BC patients and the corresponding controls. [file Image_3.jpeg]
